# Diagnostic accuracy of tumor necrosis factor-alpha assay for tuberculous pleurisy: A PRISMA-compliant meta-analysis: Erratum

**DOI:** 10.1097/MD.0000000000005950

**Published:** 2017-01-13

In the article “Diagnostic accuracy of tumor necrosis factor-alpha assay for tuberculous pleurisy: A PRISMA-compliant meta-analysis”,^[1]^ which appeared in Volume 95, Issue 48 of *Medicine*, Dr. Saeed's name was misspelled. The correct name is Ummair Saeed.
